# The impact of tailored diabetes registry report cards on measures of disease control: a nested randomized trial

**DOI:** 10.1186/1472-6947-11-12

**Published:** 2011-02-17

**Authors:** Henry H Fischer, Sheri L Eisert, M Josh Durfee, Susan L Moore, Andrew W Steele, Kevin McCullen, Katherine Anderson, Lara Penny, Thomas D Mackenzie

**Affiliations:** 1 Community Health Services, Denver Health and Hospital Authority, 777 Bannock St., Denver, 80204, USA; 2 University of Colorado Denver School of Medicine, University of Colorado Denver, 13001 E. 17thPlace, Aurora, 80045, USA; 3 Colorado School of Public Health, University of Colorado Denver, 13001 E. 17thPlace, Aurora, 80045, USA; 4 Health Services Research, Denver Health and Hospital Authority, 777 Bannock St., Denver, 80204, USA; 5 Quality Improvement, Denver Health and Hospital Authority, 777 Bannock St. Denver, 80204, USA

## Abstract

**Background:**

Most studies of diabetes self-management that show improved clinical outcome performance involve multiple, time-intensive educational sessions in a group format. Most provider performance feedback interventions do not improve intermediate outcomes, yet lack targeted, patient-level feedback.

**Methods:**

5,457 low-income adults with diabetes at eight federally-qualified community health centers participated in this nested randomized trial. Half of the patients received report card mailings quarterly; patients at 4 of 8 clinics received report cards at every clinic visit; and providers at 4 of 8 clinics received quarterly performance feedback with targeted patient-level data. Expert-recommended glycemic, lipid, and blood pressure outcomes were assessed. Assessment of report card utility and patient and provider satisfaction was conducted through mailed patient surveys and mid- and post-intervention provider interviews.

**Results:**

Many providers and the majority of patients perceived the patient report card as being an effective tool. However, patient report card mailings did not improve process outcomes, nor did point-of-care distribution improve intermediate outcomes. Clinics with patient-level provider performance feedback achieved a greater absolute increase in the percentage of patients at target for glycemic control compared to control clinics (6.4% vs 3.8% respectively, Generalized estimating equations Standard Error 0.014, p < 0.001, CI -0.131 - -0.077). Provider reaction to performance feedback was mixed, with some citing frustration with the lack of both time and ancillary resources.

**Conclusions:**

Patient performance report cards were generally well received by patients and providers, but were not associated with improved outcomes. Targeted, patient-level feedback to providers improved glycemic performance. Provider frustration highlights the need to supplement provider outreach efforts.

**Trial Registration:**

ClinicalTrials.gov: NCT00827710

## Background

There is a call for individualization of diabetes outcome targets [1,2] given potential cardiovascular harm in aggressive risk factor modification in subgroups of diabetic patients observed the ACCORD, ACCORD-BP, and INVEST studies [3-5] At the same time, most diabetic patients at our institution and nationwide fall well short of diabetes targets [6] recommended by the American Diabetes Association (ADA, [7]), the Joint National Committee on Prevention, Detection, Evaluation, and Treatment of High Blood Pressure (JNC-7, [8]), and the National Cholesterol Education Program (NCEP, [9]). Given that prospective interventional studies, including the UKPDS, VADT, ADVANCE, and Kumamoto studies [10-13], indicate improved micro- and/or macrovascular outcomes in the majority of diabetic patients that meet these targets, how can health care institutions like ours with over 7,000 diabetic patients assist patients in meeting these outcome goals?

Reviews on the impact of diabetes disease management strategies conclude that most interventions do not improve glycemic, lipid, and blood pressure performance. In particular, provider audit and feedback did not impact glycemic control [14,15] The most effective interventions are targeted and utilize case management, expansion of clinic team member roles, and/or patient self-management initiatives [14-20].

Many studies of diabetes self-management programs demonstrate improved glycemic control, and, in some cases, better lipid and blood pressure performance and less medication use. Systematic reviews highlight key features and the complexity of published self-management programs: 1) Most high-quality studies involved more than 7 hours of contact with the patient, although similar results were obtained in programs with as little as 3 hours of contact; 2) The impact on clinical outcome performance extinguishes with time, but can be maintained through refresher education; 3) Programs were delivered by health professionals trained in self-management diabetes education and little is published on the impact of self-management support provided through lay health workers [18-20].

This intervention utilizes a computerized diabetes registry to disseminate a patient self-management tool, the patient report card (PRC, Additional File 1), by mail and at the point-of-care, and to distribute provider performance report cards (PrRC), which include feedback of provider-specific data on individual patients not meeting recommended targets. The PRC includes a summary of present and past patient outcome performance and asks the patient to choose an area for self-management. We sought to answer three questions through this investigation: 1) Can we impact process outcomes by mailing the PRC to patients? 2) Can we impact clinical outcomes by promoting self-management through the patient's provider and medical assistants at each clinic visit? and 3) Can provider feedback detailing patients falling short on recommended goals spur provider and/or clinic-driven outreach that improves clinical outcomes? Given the inability to supply time-intensive diabetes self-management education to our population of over 7000 diabetic patients, we explore whether the use of health information technology and reinforcement of self-management and patient-centered care by medical assistants and providers at each clinic visit improves diabetes outcome performance. In addition, we investigated whether the use of health information technology to deliver automated, targeted patient-level feedback improves outcomes.

## Methods

### Design Overview

**Figure 1:**
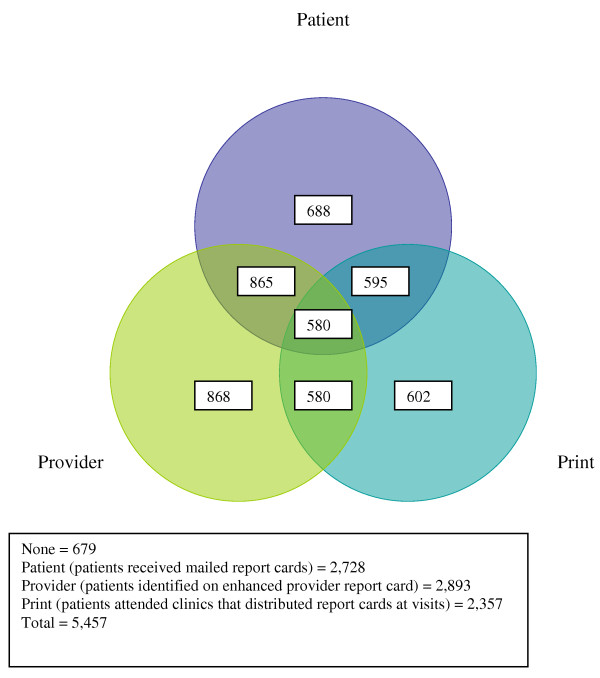
**Venn diagram of patient assignments to each of the 3 interventions**.

The intervention took place over 13 months at eight federally qualified community healthcare centers within Denver Health (DH), an urban safety-net healthcare system, ending January 1, 2009. A prospective randomized controlled design was used. Randomization for the mailed PRC took place at the patient level across the entire diabetes registry. Randomization took place at the clinic level in a 2 × 2 factorial design for the point-of-care PRC and the PrRC arms, stratified by clinic size. A given clinic was randomized to i) automated distribution of the point-of-care PRC or no distribution of the point-of-care PRC and ii) distribution of either a standard PrRC or an enhanced PrRC, which also included targeted patient level data. Of our eight clinics, 4 are relatively small and 4 are relatively large in size. We randomly assigned 1 large and 1 small clinic to each of the four design arms. Thus, a patient was randomized to one of eight possible intervention arms (Figure 1). The Colorado Multiple Institutional Review Board (COMIRB) approved this study prior to implementation. Review of our protocol by COMIRB concluded that a waiver of individual informed consent was appropriate due to the intervention being both of low risk to the patients and in accordance with quality improvement practices. In addition, the instructions provided with the self-administered survey included a clear statement that patient participation was at the patient's own discretion and was not in any way required.

### Setting and Participants

DH serves a diverse population of over 7,000 diabetic patients, many of whom are uninsured (43%), on Medicaid (18%) or Medicare (26%). Over half are Latino (59%), with a substantial African-American minority (21%). Consistent with national trends, the majority of our diabetic patients do not meet all of the targets of care recommended by the ADA, JNC7, and NCEP [7-9].

Since 2000, DH has participated through the Health Services and Resources Administration in a diabetes collaborative, which sparked our development of a computerized diabetes registry with a software interface for querying outcome performance. These tools facilitated the design and implementation of this intervention.

### Randomization

**Figure 2:**
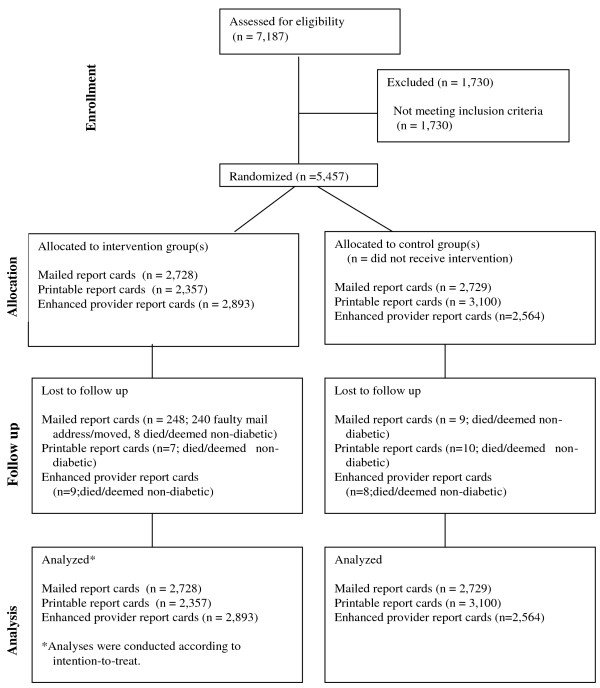
**Consort flow diagram**.

Our diabetes registry contains more than 7,000 patients over age 17 who have had i) at least one visit to a DH primary care clinic within 18 months and ii) an ICD-9 code of 250.xx, indicating diabetes, in one of those visits. To ensure a broad intervention reach, we minimized exclusion criteria to patients older than 75 years, those without a valid mailing address, and those whose primary language was neither English nor Spanish. A total of 5,457 patients were randomized to this study (Figure 2).

Using a clock-generated seed, SAS Enterprise Guide software version 9.1 (Cary, NC) was used to randomize half (2,728 patients) into the intervention group and half (2,729 patients) to the control group for PRC mailings.

Similar randomization methodology was used for the point-of-care PRC and the enhanced PrRC, with IDs assigned by clinic instead of by patient. Each of the eight clinics, four small and four large, were assigned to receive the point-of-care PRC, the enhanced PrRC, both, or neither. Small and large clinics were considered separately. One small and one large clinic were randomized into each of the four groups. Thus, a given patient fell into one of eight possible intervention arms, determined by the clinic randomization and the patient-level mailed report card randomization (Figure 1).

### Power Analyses

All power analyses were performed using Power Analysis and Sample Size (PASS 2008) software, with a significance level of each test (alpha) targeted at 0.05 with >90% certainty. The power analyses assessed the ability to i) detect changes in outcomes at the patient-level across the eight intervention arms and ii) detect changes at the clinic-level based on a cluster randomization analysis. These power calculations were based on a post hoc correction given that our a priori power calculation over-estimated the sample size.

### Intervention

#### Mailed Patient Report Cards

The PRC (Additional File 1) contained a brief explanation in both English and Spanish of the diabetes ABC's ("A" for HbA1c, "B" for blood pressure, and "C" for cholesterol); recent patient performance on the ABC's compared to national recommended targets; and encouragement for patients to choose a self-management goal. The mailed PRC also asked the patient to make an appointment if it had been two months or more since seeing a provider. PRC's were mailed to intervention patients (N = 2,728) on a quarterly basis. Usual care patients (N = 2,729) were not sent mailings. A staggered mailing approach was used to level resource demand, with one-third of mailing volume sent during each month in a quarter. Intervention patients were mailed four PRCs, one per quarter during the intervention. Patients who were found not to have diabetes, who died, or who relocated without a valid forwarding address were removed from the mailing list.

A brief one-page survey, developed by the project team and approved by COMIRB, was included with the second and fourth quarter PRC mailings. The survey was designed to evaluate patient satisfaction with and perceptions of the tailored report card in the areas of report card content and use, provider communication, and diabetes control. The survey was comprised of 10 items with agree-to-disagree responses on a scale from 1 to 5, one additional yes-no item, and 3 questions which invited unstructured responses. Patients were encouraged to provide their opinions, assured that all responses would be treated confidentially, and thanked for their time and participation. Surveys were self-administered and were printed in both English and Spanish. A self-addressed, postage-paid envelope was included with each survey to facilitate response; however, neither reminders nor additional incentives for completion were offered. This approach was selected to emulate patient satisfaction assessments as conducted on an ongoing, sustainable basis in a healthcare system with limited financial resources. A total of 5,359 surveys were mailed, 2,686 surveys in the second-quarter and 2,673 in the fourth quarter.

#### Point-of-Care Patient Report Cards

Distribution of the PRC at the point-of-care occurred at four randomly selected clinics where it was generated automatically during each primary care visit check-in. The point of care distribution of the PRC was tested for approximately 2 months at a clinic site that was randomly selected to continue point-of-care PRM distribution. At clinics where the Patient Report Card automatically printed at the time of registration, medical assistants, as they checked a patient in, briefly encouraged the patient to make a self-management goal, answered questions about the PRC, and/or briefly assessed the status of an already existing self-management goal. The patient was then directed to further discuss their goal with their provider. The staff received training in self-management and patient-centered care through a Certified Diabetes Educators (3 hours yearly), and this was re-enforced for staff and providers at monthly clinic-level collaborative meetings.

#### Provider Performance Report Cards: Standard and Enhanced

The standard PrRC is generated from the diabetes registry on a quarterly basis and was made available to all clinicians as part of usual diabetes care for two quarters prior to this intervention. It is published on an internal website and distributed to providers as a quarterly email containing a link to the most recent update. Archived versions of the PrRC remain accessible through the website. The PrRC includes:

• the provider's performance across his/her patient panel on intermediate outcomes (including average HbA1c, percent with HbA1c < 7.0 percent, percent with LDL < 100 mg/dl, percent with blood pressure < 130/80 mm Hg, and percent with a self-management goal)

• the mean outcome performance across all providers at a clinic

• the aggregate and individual performance of all the providers at a clinic on each intermediate outcome

• the target performance for each outcome across all clinic sites

All providers at the eight clinics continued to receive the standard PrRC throughout the intervention period. Providers randomized to the enhanced PrRC intervention received with the PrRC notification email a list of up to 10 of their patients who met certain preset criteria based on patients' HbA1c, LDL, or blood pressure not being at goal. List criteria changed quarterly and were based on the most current clinical information at the creation of the enhanced PrRC.

The components of this intervention were communicated to each clinic's designated "diabetes champion" (DC) and staff at individual clinic meetings and again to providers at system-wide provider meetings. The expectation of either provider-driven or team-driven outreach to patients identified on the enhanced PrRC was expressed, and it was left to the DC to determine the nature of the outreach at each clinic.

The DC at each clinic was also asked to consent to in-person interviews with an investigator, with all interview responses to be treated confidentially. All eight identified DCs agreed to participate. Interviews were conducted with the eight DCs twice each, at the middle of the project period and after the intervention was completed. All interviews were semi-structured, according to an interview guide developed by the project team, in order to ensure attention to key subjects while also allowing for the in-depth exploration of additional topics and areas of interest which might emerge during the interview. Interview topics included ways a provider might help patients manage diabetes, provider-level and clinic-level initiatives to improve diabetes care and intermediate diabetes health outcomes, the patient report card interventions (both mailed and on-site printed versions), and the system-wide provider performance feedback program. Audio recordings of interviews were made with DC consent, and augmented by interviewer note-taking.

### Outcomes and Measures

Process outcomes were analyzed for those patients who received mailed patient report cards compared to those who did not and include the percent of patients with one or more measurements during the intervention of a i) HbA1c ii) LDL or iii) blood pressure. We also measured intermediate outcome performance on glycemic control (% of patients with HbA1c < 7), lipids (% of patients with LDL < 100 mg/dL, and blood pressure (% of patients with BP < 130/80 mm Hg), in accordance with expert recommended guidelines of the American Diabetes Association (ADA, 7), the Joint National Committee on Prevention, Detection, Evaluation, and Treatment of High Blood Pressure (JNC-7, 8), and the National Cholesterol Education Program (NCEP, 9). Intermediate outcome analysis was done at the clinic level for the PRC (the 4 clinics with point of care PRC versus the 4 without point of care PRC) and for the PrRC (the 4 clinics with the enhanced PrRC versus the 4 with the standard PrRC). Intermediate outcome analysis was also done at the patient level across all eight intervention clinics (each patient may or may not have been randomized to the mailed PRC, the point of care PRC, and/or the enhanced PRC) as well as for the PrRC. The patient level PrRC compared all patients meeting the pre-set enhanced PrRC criteria at clinics with the enhanced PrRC versus those at clinics with the standard PrRC.

#### Patient and provider satisfaction analyses

Responses to scaled survey items were analyzed using SAS Enterprise Guide software 9.1 (Cary, NC) Evaluation of open-ended survey response and interview data was undertaken using a content analysis approach. Unstructured responses to open-ended survey questions were subjected to inductive analysis for category development. An open coding process was used to develop heuristic codes from themes and patterns that emerged during review of response data. The initial codes were then reexamined in context and refined into an objective code set, which was utilized by clinician and non-clinician reviewers for use in final coding and interpretation of survey responses. Interview data were analyzed through non-clinician investigator review and inductive analysis of written transcripts and audio recordings. Clinician review of interview data was not conducted in order to preserve provider confidentiality. Emergent themes and patterns among patients' and providers' remarks were identified and incorporated into a synthesis of results.

### Statistical Analysis

Analyses adjusted for differences in age, race/ethnicity, gender, degree of illness, and baseline levels for each outcome variable, and included generalized estimating equations (GEE) to account for the within-subject correlation of repeated measures by individual patients. The standard errors and 95% confidence intervals for the analyses of GEE are included with the *p-*values in reported results. Patients who died, changed clinics during the intervention period, or moved away were analyzed according to intention-to-treat (Figure 2). All analyses were performed using SAS Enterprise Guide software version 9.1 (Cary, NC).

The Chronic Illness and Disability Payment System (CDPS) version 2.5 was used to risk adjust for differences in the degree of illness between the control and intervention groups [21]. CDPS uses ICD-9-CM codes to group and weight diagnoses for chronic and disabling diseases.

## Results

### Baseline Characteristics

Randomization for the mailed PRC took place at the patient level and resulted in similar baseline characteristics (Additional File 2) for the control (n = 2,729) and intervention (n = 2,728) groups, except for a small but significant age difference and a nearly significant difference in gender. Randomization for the point-of-care PRC and the enhanced PrRC took place at the clinic level and resulted in similar gender and age distribution for the four groups. The groups differed significantly in race/ethnicity, as expected given the different neighborhoods served by each clinic. Patients lost to follow up in the intervention group were not significantly different in demographic make-up to those in control group and were analyzed with an intention-to-treat threshold.

### Power Analysis

The study was adequately powered to detect an improvement of between 3 and 7 absolute% for the glycemic, blood pressure, and lipid measures across all intervention arms. The cluster analyses at the clinic level did not afford adequate power to detect reasonable differences should they exist in proportions between control and intervention groups for any of the variables assessed. This is a limitation of this study discussed further in the conclusion section.

### Outcomes

#### Mailed Patient Report Card

The data suggest that patients who received the mailed report cards were in fact less likely to present for testing for blood pressure by absolute 2% (GEE SE = 0.008, p = 0.004, CI 0.0074 - 0.0396) and glycemic control by absolute 3% (GEE SE = 0.009, p < 0.001, CI 0.0131 - 0.0495) than those in the control group. Testing for lipids was not significantly different between the two groups. A sub-analysis was performed to determine whether a patient's performance on his/her PRC was associated with an effect on process outcomes; the results were similar to the overall findings.

A total of 5,359 surveys were mailed over the course of the study. Of that number, 349 completed surveys were returned, 198 in the second quarter and 151 in the fourth quarter, for an overall response rate of 6.51%. Results of scaled-item analysis have therefore not been included in quantitative analyses intended for generalization to the population level, but have been considered supplemental to qualitative data obtained through content analysis of unstructured, patient-provided feedback. Most respondents expressed overall satisfaction with the design, usability, and content of the mailed report cards and indicated a wish to continue receiving them.

#### Point-of-Care Patient Report Card

**Figure 3:**
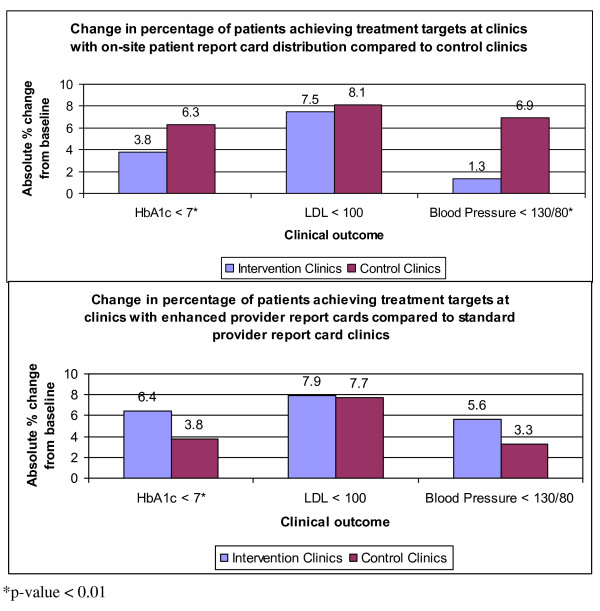
**Outcomes at clinics for point of care patient and provider reports**.

Patients who received the PRC at the point-of-care were not significantly different from controls on the lipid target (LDL < 100 mg/dL) but performed worse on glycemic (HbA1c < 7) and blood pressure (BP < 130/80 mmHg) targets (Figure 3 top panel). For glycemic control, 29.8% of controls were at goal at baseline versus 36.1% post-intervention, as compared to 30.7% at baseline and 34.5% post-intervention for those receiving the point-of-care PRC (GEE SE 0.013, p = 0.001, CI 0.017 - 0.068). For blood pressure control, 43.2% of controls achieved the blood pressure target at baseline versus 50.1% post-intervention, as compared to 38.3% at baseline and 39.6% post-intervention for those receiving the point-of-care PRC (GEE SE 0.016, p < 0.001, CI 0.034 - 0.080).

#### Enhanced Provider Report Card

**Figure 4:**
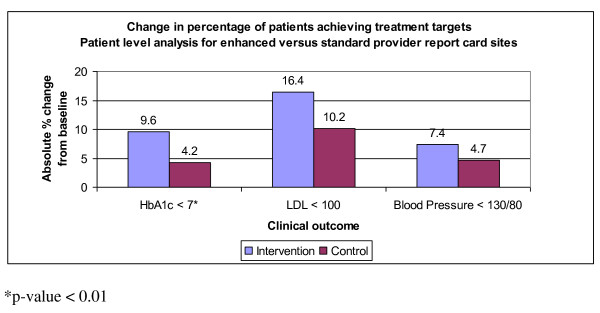
**Patient level analysis for enhanced versus standard provider report card sites**.

Patients at clinics with the enhanced PrRC had significantly greater absolute percent increase in glycemic control compared with patients at clinics with the standard PrRC (6.4% versus 3.8% respectively, GEE SE 0.014, p < 0.001, CI -0.131 - -0.077). Absolute percent improvements in lipid and blood pressure control at the enhanced PrRC sites (7.9% and 5.6% respectively) compared to the standard PrRC sites (7.7% and 3.3% respectively) were not statistically different (Figure 3 lower panel). In the patient-level analysis, compared to matched controls, patients on the PrRC improved an additional absolute 5.4% on the glycemic target, 6.2% on the lipid target, and 2.7% on the blood pressure target (Figure 4); however, glycemic performance was again the only measure that achieved statistical significance (GEE SE 0.012, p < 0.001, CI 0.019 - 0.067).

Perception among providers who received enhanced feedback with patient-level data was that the list of "enhanced-focus" patient names contained too few items and was not sent frequently enough. In addition, concurrent initiatives were identified at two of the intervention clinics that involved generating separate, more frequent, and more inclusive lists of patients and distributing them to providers for action, which may have affected the results of the enhanced feedback effort. The performance feedback reports were also seen to promote a sense of competition among providers and clinics. Reactions to such competition were mixed. Provider frustration with a perceived inability to affect change was also mentioned as a concern, stemming from a perception that providers are already aware of the issues but lack adequate resources to address them. Specific examples of desired resources include additional personnel for education and case management as well as the equipment and materials needed to conduct HbA1c testing at the point of care.

#### Patients with Multiple Interventions

**Figure 5:**
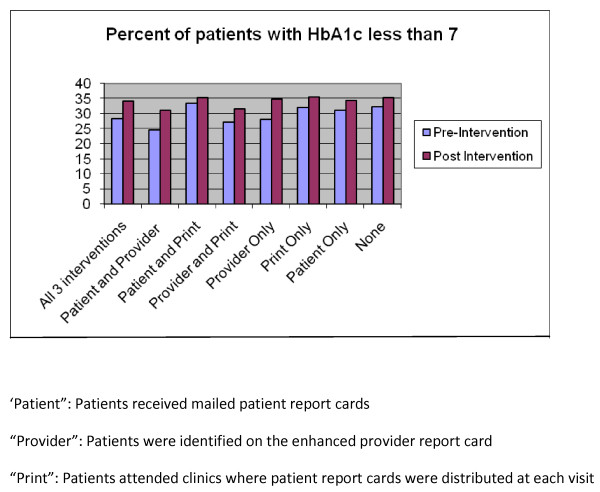
**Analysis at the patient level across all 3 interventions**.

Patients at two clinics received both the point-of-care PRC and were assigned to providers receiving the enhanced PrRC. These patients performed the same on glycemic and lipid measures but worse on blood pressure control than patients at the two clinics receiving neither of these interventions (data not shown). These intervention patients had an 0.5% absolute increase in blood pressure control (< 130/80 mm Hg) compared to a 3.8% absolute increase among control patients (GEE SE 0.009 p = 0.041, CI 0.002 - 0.073). In addition, analysis was performed at the patient level, comparing the eight different intervention arms, made up of the 2 × 2 PRC and PrRC design combined with patient PRC mailings. There was no difference in process or intermediate glycemic, lipid, and blood pressure outcomes performance between patients that received all 3 interventions (mailed PRC, point-of-care PRC, and providers with enhanced PrRC) and those who received either none, one, or two of the 3 possible interventions (See Figure 5 for display of glycemic analysis).

## Conclusion

We successfully automated the PRC mailing process through a monthly query of our diabetes registry to generate customized report cards and mailing labels across an intervention group of 2,729 patients. Ultimately, the mailings did not improve process outcomes as compared to an equally-sized usual care group. Given the potential cost of quarterly mailings to all patients with diabetes, this finding is of great value to organizations with large registries. While this finding allows redistribution of resources away from patient mailings, it leaves unanswered the question of how to improve process outcomes. One reason for the lack of effect might include our reliance on paper-based communication with much text and few graphics to a population known to have lower literacy.

Patients who received the point-of-care PRC performed similarly to control patients on lipids, but significantly worse than control patients on glycemic and blood pressure targets (Figure 3 top panel). As this finding is counterintuitive to the nature of chronic disease management, in that provision of health information at the point-of-care should not cause worse clinical outcome performance, it points to the limitations inherent in studying the impact of chronic disease management programs in a large, diverse healthcare system. Innate differences exist among patient populations and clinics, even within the same safety-net healthcare system. Attempts can be made to control for these differences among variables in statistical models but may fail to incorporate social and contextual factors, such as community-based influences or the motivational impact of a clinic leader. Another significant challenge is in accounting for the effect of concomitant quality improvement efforts. Clinics were not discouraged from pursuing their own initiatives during this study period, and numerous programs were found to have been concurrently implemented, including: i) distribution of additional at-risk patients; ii) a blood pressure management initiative; iii) lipid and glycemic nurse case management; iv) further self-management promotion; and v) a system-wide medication reconciliation program. While these diverse interventions contributed to intermediate outcome improvement across the entire patient cohort and to a relatively greater impact on blood pressure performance in the usual care group, they also complicate the final analysis. The negative results also call into question whether frequent, but brief, self-management support through medical assistants and providers at the point of care is an effective means of supporting patient self-management.

Although the point-of-care PRC did not improve clinical outcomes, both patients and providers expressed satisfaction with its potential to motivate behavioral change. Given the positive feedback and the automation that facilitated point-of-care distribution with minimal resource utilization, we have elected to disseminate an enhanced version of the point-of-care PRC to all 8 clinics.

The enhanced PrRC was associated with significant improvement in glycemic control at the clinic level (figure 3 bottom panel) as well as at the patient level (figure 4). However, providers expressed frustrations with performance feedback that corroborated recent expert panel discussions [23,24]. In short, the many competing aims in the traditional 20-minute visit often leave both patient and provider unsatisfied. Rarely does the healthcare system engage patients between visits in order to identify barriers, assess performance, and help manage medicines. The enormous potential of a team-based between-visit management approach involving ancillary staff and nurses and a variety of modalities, such as cell phones, text messaging, and email, is not yet fully tapped. The current intervention confirms that we can automate a diabetes registry and improve care by delivering targeted patient-specific data. We anticipate greater clinical impact and improved patient and provider satisfaction as we incorporate health information delivery into novel modes of team-based primary care.

There are several other limitations to our study not mentioned above. Despite randomization, race/ethnicity varied significantly between the clinic groupings given the different demographics around our individual clinics; we controlled for these differences in our regression models. An additional limitation to our study design is the lack of power to detect achievable performance changes at the clinic level. Unfortunately, it is sometimes not feasible to implement disease management programs at the provider or patient level, such as automated point of care patient report card distribution. In attempt to achieve sufficient statistical power, analysis was also done at the patient-level across the eight potential intervention groups and showed no significant difference for glycemic, lipid, and blood pressure measures. Although the 6.5% survey response rate was in-line with our expectations for a non-incentivized mail-in survey in a safety net population, it did not allow for quantitatively generalizable statistical analyses. Finally, the study protocol was not published prior to the initiation of the study which limits the readers' ability to ascertain adherence to a pre-established study design.

## Competing interests

The authors declare that they have no competing interests.

## Authors' contributions

The manuscript was drafted by HF. All authors contributed to discussions of the designs of the study, analysis and interpretation of the data, and critical review of the manuscript. All authors read and approved of the final, submitted manuscript.

## Authors' Information

HF: Practicing internist and director of the Denver Health Diabetes Collaborative

SE: PhD in Economics and conducts health services research at the Denver VA Medical Center

MJD Research project coordinator and quantitative data analyst

SM Assistant director of health services research at Denver Health Medical Center

AS Practicing Internist and Director of Medical Informatics at Denver Health Medical Center

KM Application analyst for chronic disease data warehouse at Denver Health Medical Center

KA Practicing family practitioner with interest in health care maintenance research

LP Practicing family practitioner with interest in Quality Improvement

TM Chief Quality Officer at Denver Health Medical Center and health services researcher

## Pre-publication history

The pre-publication history for this paper can be accessed here:

http://www.biomedcentral.com/1472-6947/11/12/prepub

## Supplementary Material

Additional file 1 **Patient report card**. Sample patient report cared Click here for file

Additional file 2 **Patient demographics for mailed report cards, point of care patient report cards, and enhanced provider report cards**. Demographics Click here for file
