# Supplementary material for: The impact of tailored diabetes registry report cards on measures of disease control: a nested randomized trial
Source: BMC Med Inform Decis Mak. 2011 Feb 17;11:12. doi: 10.1186/1472-6947-11-12 (PMC3050679; doi:10.1186/1472-6947-11-12)
Supplement: Additional file 2 — Patient demographics for mailed report cards, point of care patient report cards, and enhanced provider report cards. Demographics [file 1472-6947-11-12-S2.DOC]

|  |  | **Patient Mailed Report Cards*** | | | **Point of Care Patient Report Cards^†^** | | | **Enhanced Provider Report Cards^†^** | | |
| --- | --- | --- | --- | --- | --- | --- | --- | --- | --- | --- |
|  | All patients | Intervention | Control | *p-value* | Intervention | Control | *p-value* | Intervention | Control | *p-value* |
| **Demographics** | N=5,457 | N = 2,728 | N = 2,729 |  | N=2,357 | N=3,100 |  | N=2,893 | N=2,564 |  |
| **Mean Age (SD)** | 54.1 | 54.4 (11.9) | 53.8 (12.0) | 0.05 | 54.3 (11.9) | 54.0 (12.1) | 0.36 | 54.2 (11.9) | 54.0 (12.1) | 0.53 |
| **Gender** |  |  |  | 0.09 |  |  | 0.14 |  |  | 0.07 |
| Female | 59.3 | 58.1 | 60.4 |  | 58.4 | 60.4 |  | 60.4 | 58 |  |
| Male | 40.7 | 41.9 | 39.6 |  | 41.6 | 39.6 |  | 39.6 | 42 |  |
| Total | 100 | 100 | 100 |  | 100 | 100 |  | 100 | 100 |  |
| **Race/Ethnicity** |  |  |  | 0.46 |  |  | <0.01 |  |  | <0.01 |
| Asian | 0.7 | 0.7 | 0.6 |  | 0.4 | 0.8 |  | 0.7 | 0.6 |  |
| African American | 15.9 | 15.3 | 16.5 |  | 24.7 | 9.2 |  | 15.6 | 16.3 |  |
| Hispanic | 61.5 | 61.6 | 61.4 |  | 58.6 | 63.7 |  | 64 | 58.7 |  |
| White | 16.9 | 17 | 16.9 |  | 10.9 | 21.6 |  | 14.5 | 19.7 |  |
| Unknown | 5 | 5.4 | 4.6 |  | 5.4 | 4.7 |  | 5.2 | 4.7 |  |
| **Total** | 100 | 100 | 100 |  | 100 | 100 |  | 100 | 100 |  |

*Randomized at patient level

†Randomized at clinic level
