# Supplementary material for: The impact of tailored diabetes registry report cards on measures of disease control: a nested randomized trial
Source: BMC Med Inform Decis Mak. 2011 Feb 17;11:12. doi: 10.1186/1472-6947-11-12 (PMC3050679; doi:10.1186/1472-6947-11-12)
Supplement: Supplementary file 2 [file 1472-6947-11-12-S1.DOC]

DENVER HEALTH
Level One Care for ALL

Your Diabetes Report Card

106900772 11 22
HSC: TPO
TEST ,TESTKUJO
2287647 F 02/28/1950
CICP DVR:
1: 20 30 RX:07 IP: 235
PCP: SHLAY, JUDITH C
HPHN:555 555-5555 MRLO:

Do you know your **Diabetes ABC'S Report Card Numbers**?

A is for A1C. This test measures your average blood sugar (glucose) over the last 3 months.

B is for Blood Pressure. High blood pressure makes your heart work too hard and can damage it.

C is for Cholesterol. Bad cholesterol, or LDL, builds up and clogs the blood vessels to your heart.

S is for Self –Management Goal - What you are going to do to help improve your Diabetes control ?

Bad news: If your **ABC'S** are bad, you are at higher risk for heart attack, stroke, kidney disease, and blindness. With Diabetes, Heart disease is more likely to strike you at an earlier age than someone without diabetes.

Good news: You can fight back! Practicing good health behaviors will help you to take control of your Diabetic **ABC'S** and help you to live a longer and healthier life !

Here is your personal **Diabetes ABC'S Report Card:**

	Your Most Recent Result	Your Previous Result	Next Test Due On	
A = A1c<br>Goal is less than 7	**Excellent** 5.9 09/20/07	**Excellent** 6 02/27/07	PAST DUE	Excellent
**B = Blood Pressure**<br>**Goal is 130/80 or less**	**Fair** 120/80 07/15/08	**Fair** 120/80 07/11/08	10/15/08	Fair<br>Poor
**C = Cholesterol - LDL**<br>**Goal is less than 100**	**Fair** 107 06/15/08	**Excellent** 67 02/08/08	09/15/08	Very Poo

S = Self-Management Goal: Your goal is XELF MANAGEMENTXXXXXXXX XXXXXXXXX XXXXXXX XXXXXXX XXXXXXX!.

Your risk for having a Heart Attack in the next 10 years based on your current ABC'S Report Card is:
18% / MODERATE RISK

Basic Diabetes Education Lessons: You have completed 4 of the seven (7) recommended lessons:
1. Basic Pack 03/31/08 2. Diabetes Video 03/04/08 3. Diabetes Plan ______
4. Self-Management Goal Set 07/01/08 5. Basic Nutrition with Nurse ______
6. Nutrition Video ______ 7. Learning Assessment 04/09/08

My Action Plan:

If you are not at your goal for one or more of your ABC'S above or do not have a Self-Management Goal, consider choosing one of the self-management goals listed below to help you reach your healthy goals.

☐ Making and keeping more regular appointments – Ask your provider how many visits you need.

☐ Taking my medications exactly as prescribed. What one specific thing will you do to be sure you take your medications as prescribed? ______________________________

____

☐ Improving my eating habits. What one specific thing will you change?

____

☐ Increasing my Physical Activity:

I will ______________ (activity) ______ times per week for ______ minutes each time.

Bring this form with you to your next visit to discuss your plans and your goals with your provider.
